# *NR4A2* expression is not altered in placentas from cases of growth restriction or preeclampsia, but is reduced in hypoxic cytotrophoblast

**DOI:** 10.1038/s41598-021-00192-y

**Published:** 2021-10-19

**Authors:** Natasha de Alwis, Sally Beard, Natalie K. Binder, Natasha Pritchard, Tu’uhevaha J. Kaitu’u-Lino, Susan P. Walker, Owen Stock, Katie M. Groom, Scott Petersen, Amanda Henry, Joanne M. Said, Sean Seeho, Stefan C. Kane, Stephen Tong, Natalie J. Hannan

**Affiliations:** 1 Therapeutics Discovery and Vascular Function in Pregnancy Group, Mercy Hospital for Women, Heidelberg, VIC 3084 Australia; 2 Translational Obstetrics Group, Mercy Hospital for Women, Heidelberg, VIC 3084 Australia; 3 Mercy Perinatal, Mercy Hospital for Women, Heidelberg, VIC 3084 Australia; 4 Department of Obstetrics and Gynaecology, University of Melbourne, Mercy Hospital for Women, 163 Studley Rd, Heidelberg, VIC 3084 Australia; 5 Liggins Institute, University of Auckland, Auckland, 1023 New Zealand; 6 Centre for Maternal Fetal Medicine, Mater Mothers’ Hospital, South Brisbane, QLD 4101 Australia; 7 School of Women’s and Children’s Health, UNSW Medicine, University of New South Wales, Sydney, Australia; 8 Maternal Fetal Medicine, Joan Kirner Women’s and Children’s Sunshine Hospital, St Albans, VIC 3021 Australia; 9 Northern Clinical School, Women and Babies Research, The University of Sydney, St Leonards, NSW 2065 Australia; 10 Department of Maternal Fetal Medicine, Royal Women’s Hospital, Parkville, VIC 3052 Australia

**Keywords:** Intrauterine growth, Gene expression, Predictive markers, Genetics research

## Abstract

Nuclear Receptor Subfamily 4 Group A Member 2 (*NR4A2*) transcripts are elevated in the circulation of individuals whose pregnancies are complicated by preterm fetal growth restriction (FGR). In this paper, we show that the cases with preeclampsia (PE) have increased circulating *NR4A2* transcripts compared to those with normotensive FGR. We aimed to establish whether the dysfunctional placenta mirrors the increase in *NR4A2* transcripts and further, to uncover the function of placental *NR4A2. NR4A2* expression was detected in preterm and term placental tissue; expressed higher at term. *NR4A2* mRNA expression and protein were not altered in placentas from preterm FGR or PE pregnancies. Hypoxia (1% O_2_ compared to 8% O_2_) significantly reduced cytotrophoblast *NR4A2* mRNA expression, but not placental explant *NR4A2* expression. Silencing cytotrophoblast *NR4A2* expression under hypoxia (via short interfering (si)RNAs) did not alter angiogenic Placental Growth Factor, nor anti-angiogenic *sFlt-1* mRNA expression or protein secretion, but increased expression of cellular antioxidant, oxidative stress, inflammatory, and growth genes. *NR4A2* expression was also not altered in a model of tumour necrosis factor-α-induced endothelial dysfunction, or with pravastatin treatment. Further studies are required to identify the origin of the circulating transcripts in pathological pregnancies, and investigate the function of placental *NR4A2.*

## Introduction

Preeclampsia and fetal growth restriction affect around 10% of pregnancies worldwide and cause significant maternal and perinatal morbidity and mortality^1–3^. Furthermore, exposure of a child to an adverse gestational environment is associated with increased risk of permanent neurodevelopmental, cardiovascular and metabolic impairment that persists into adulthood^4^, and preeclampsia is associated with compromised long-term maternal health^5^. Unfortunately, we are still unable to predict these conditions accurately, and have limited treatment options available. Improving methods of prediction, diagnosis, and treatment is paramount to improving patient outcomes.

Placental dysfunction is central to the development of both preeclampsia and fetal growth restriction. A healthy pregnancy largely relies on a well-developed and functional placenta, facilitating exchange of nutrients, oxygen, and waste products between the maternal and fetal systems^6^. Placental dysfunction is associated with impaired maternal uterine spiral artery remodelling, resulting in a high resistance, pulsatile blood supply. This compromised blood supply restricts nutrient and oxygen delivery to the fetus and causes oxidative stress, fluctuating oxygen tensions, and ischemia^7,8^, thereby impacting fetal growth. Early identification of placental dysfunction may help detect high-risk pregnancies that require additional monitoring of fetal wellbeing.

Multiple protein biomarkers have been proposed for early detection of fetal growth restriction, but to date, none have sufficient accuracy to be recommended for clinical practice^9,10^. Alternatively, characterising circulating levels of nucleic acid transcripts released by the placenta during pregnancy may offer a diagnostic option for detecting pregnancies affected by placental dysfunction^11,12^. In the Fetal OXygenation (FOX) Study, we identified altered mRNA transcript levels in the circulation of women whose pregnancies were complicated by preterm fetal growth restriction, associated with placental insufficiency^13^. One of the genes identified to be most highly altered was Nuclear Receptor Subfamily 4 Group A Member 2 (*NR4A2*; also known as *NURR1*). *NR4A2* is a transcription factor and early response gene, and has been implicated in many pathways and diseases including inflammation and cell proliferation^14–21^, intestinal regeneration after ischemic/reperfusion injury^22,23^, liver fibrosis^24^, cancer^19,25–27^, impaired neurodevelopment and autism^28–30^, Parkinson’s disease^31–33^, and cardiovascular disease^34–36^.

Although we identified increased *NR4A2* transcripts in the maternal circulation of pregnancies complicated by fetal growth restriction, the previous paper did not identify whether the transcripts originated from the dysfunctional placenta. This paper aims to identify whether the altered *NR4A2* transcript levels in the maternal circulation of pregnancies complicated by fetal growth restriction had associations with preeclampsia, and whether the elevated circulating *NR4A2* transcripts were reflected in the dysfunctional and/or hypoxic placenta. Furthermore, this paper attempts to identify the role of *NR4A2* in the placenta.

## Results

### Circulating *NR4A2* mRNA levels are increased in individuals with coexistent preeclampsia and fetal growth restriction

**Figure 1:**
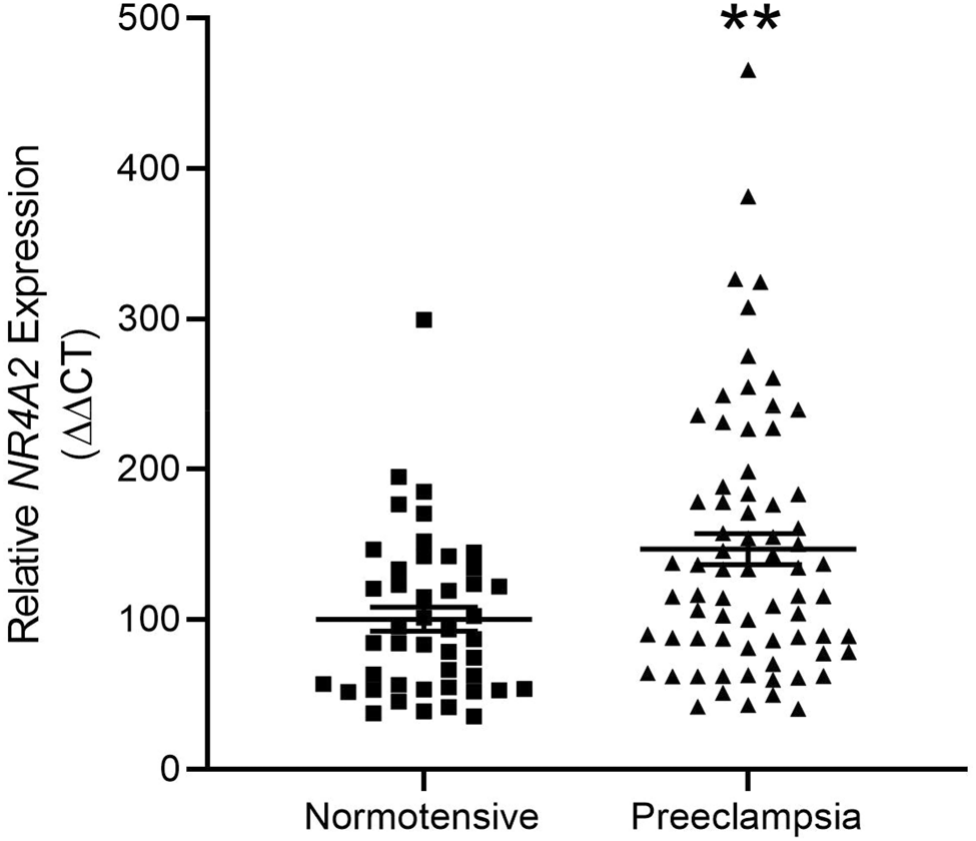
Circulating *NR4A2* mRNA in cases of normotensive preterm fetal growth restriction and preeclampsia with growth restriction (< 34 weeks gestation). RNA levels were assessed by qPCR. Cases with preeclampsia and growth restriction had significantly higher circulating *NR4A2* mRNA compared to normotensive cases of growth restriction. Data presented as relative change from normotensive levels; mean ± SEM. **p < 0.01. Normotensive, n = 45; preeclampsia, n = 71.

Using next generation sequencing, we previously reported increased *NR4A2* transcripts in the circulation of women whose pregnancies were complicated by preterm fetal growth restriction (< 34 weeks gestation)^13^. In the current study, we performed a sub-analysis, directly comparing cases with preterm preeclampsia and growth restriction to cases of normotensive growth restriction. We found significantly increased circulating *NR4A2* transcripts in the growth restricted cases with preeclampsia, compared to normotensive cases (Fig. 1).

### Placental *NR4A2* expression is increased at term

**Figure 2:**
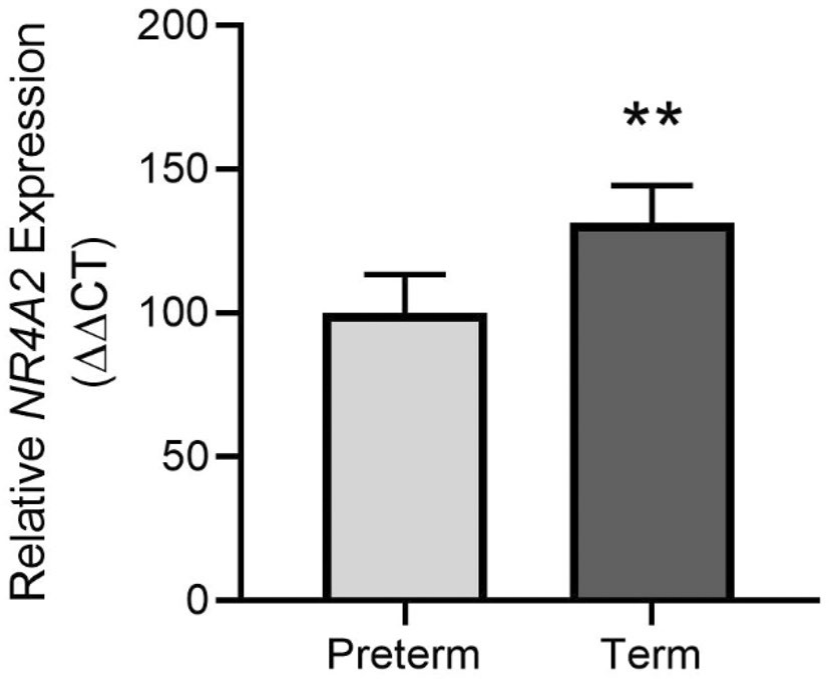
*NR4A2* expression in preterm and term placental tissue assessed by qPCR. Term placental tissue had significantly higher *NR4A2* mRNA expression compared to preterm tissue. Data presented as relative change from preterm levels; mean ± SEM. **p < 0.01. Preterm n = 30, 24–36 weeks; term n = 29, 37–41 weeks.

*NR4A2* expression was detected in all placental tissues collected, preterm (24–36 weeks) and term (37–41 weeks). *NR4A2* expression was higher in term placental tissue compared to preterm placental tissue (Fig. 2).

### *NR4A2* expression is not altered in preterm pathological placental tissue

**Figure 3:**
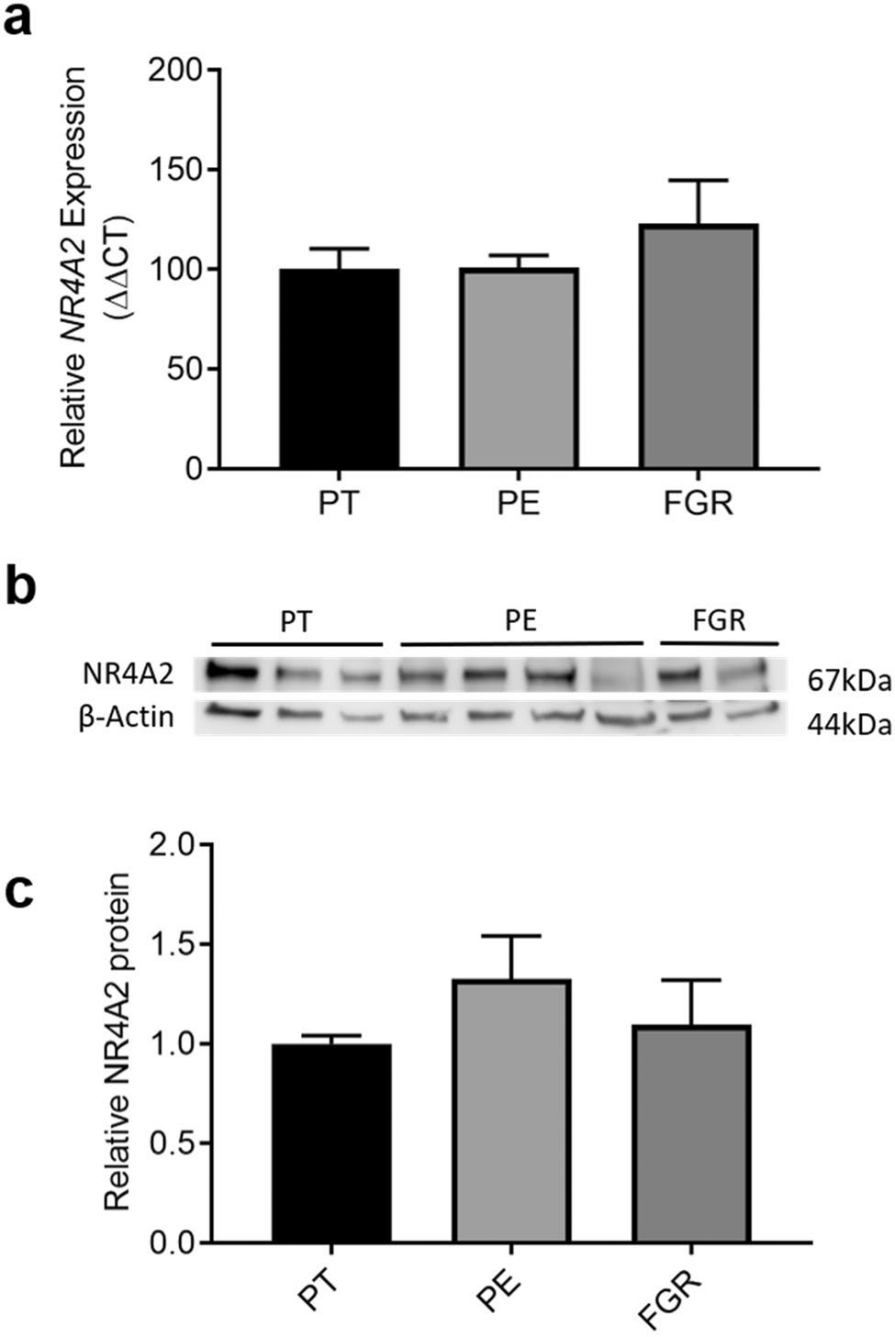
*NR4A2* expression and protein in preterm control (PT), preeclamptic (PE) and fetal growth restricted (FGR) placental tissue (≤ 34 weeks gestation). (**a**) mRNA expression assessed by qPCR. (**b**) Representative western blot. (**c**) Densitometric analysis of western blot. *NR4A2* expression was not altered in the PE and FGR placental tissue compared to respective PT control tissue (PT n = 10, PE n = 49, FGR n = 14). There was no significant difference in NR4A2 protein between the pathological and control tissue (PT n = 15, PE n = 31, FGR n = 17). β-actin acted as the loading control. Data presented as relative change from preterm controls ± SEM.

To elucidate whether *NR4A2* has a role in placental dysfunction, we investigated *NR4A2* expression in placenta collected from pregnancies complicated by preterm preeclampsia and fetal growth restriction (≤ 34 weeks gestation), conditions that feature impaired placental development and dysfunction. There was no significant difference in *NR4A2* expression in placental tissue from pregnancies complicated by either preterm fetal growth restriction or preeclampsia (≤ 34 weeks gestation) compared to preterm control tissue (Fig. 3a). NR4A2 protein levels were also not altered in the preterm pathological tissue compared to preterm controls (Fig. 3b,c; Supplementary Fig. S1). *NR4A2* mRNA expression and protein levels were not significantly altered by fetal sex in preterm pathological placental tissue or controls (data not shown).

### *NR4A2* expression is decreased in cytotrophoblast, but not placental explants under hypoxia

**Figure 4:**
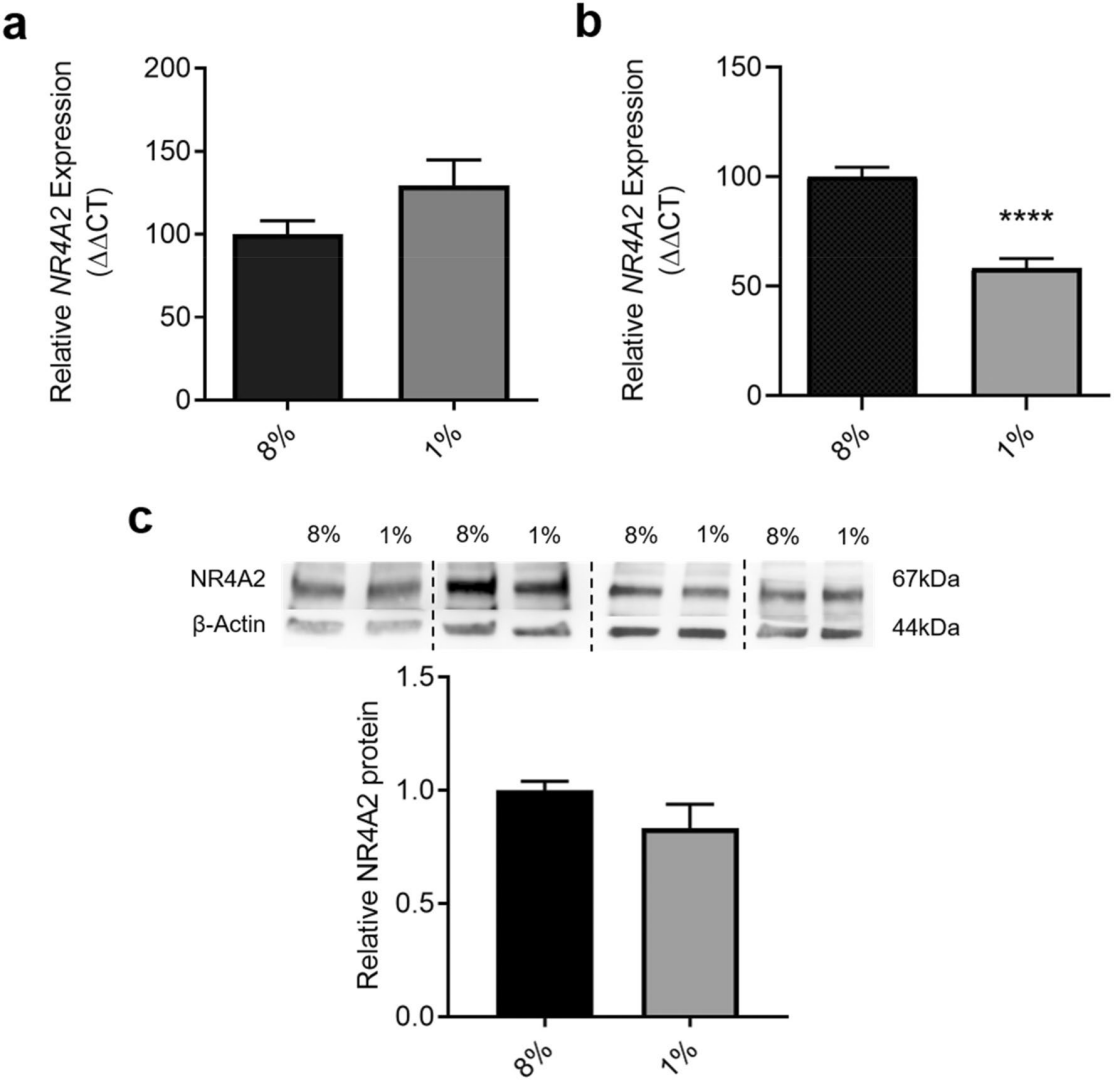
*NR4A2* expression in placental explant tissue and primary cytotrophoblast under normoxic (8% O_2_) and hypoxic (1% O_2_) conditions. *NR4A2* expression was unaltered in placental explant tissue with hypoxia (**a**). In primary cytotrophoblasts, *NR4A2* expression was significantly decreased under hypoxic conditions (**b**). There was no change in NR4A2 protein production with hypoxia (**c**). Data presented as fold change from control ± SEM. qPCR: n = 4–5 experimental replicates, each sample from a different patient. Each sample was run in triplicate. Western blot: n = 4 experimental replicates, with each sample from a different patient. Each experiment was run in triplicate and replicate lysates were pooled. β-actin acted as the loading control. ****p < 0.0001.

To further elucidate the role of *NR4A2* in the placenta, its expression was assessed in placental explants and cytotrophoblast, cells unique to the placenta. These cells and tissues were exposed to hypoxia to simulate a low oxygen environment akin to that in placental insufficiency. *NR4A2* expression was detectable in both placental explant tissue and primary cytotrophoblast. There was no difference in *NR4A2* expression in placental explants under hypoxia compared to normoxic conditions (Fig. 4a). In cytotrophoblast, *NR4A2* expression was significantly reduced under hypoxia (*p* < 0.0001; Fig. 4b). However, there was no significant change in cytotrophoblast NR4A2 protein levels in hypoxic compared to normoxic conditions (Fig. 4c; Supplementary Fig. S2).

### Knockdown of *NR4A2* expression in primary cytotrophoblast

In primary cytotrophoblast, *NR4A2* expression was significantly reduced (> 50%) with addition of *NR4A2* targeting siRNAs compared to the negative control in both normoxic (*p* = 0.0028; Supplementary Fig. S3a) and hypoxic conditions (*p* = 0.0002; Supplementary Fig. S3b). Silencing *NR4A2* did not impact cell survival under either normoxic or hypoxic conditions (Supplementary Fig. S3c,d).

### Silencing *NR4A2* in cytotrophoblasts does not alter expression or secretion of sFLT-1 or PGF

**Figure 5:**
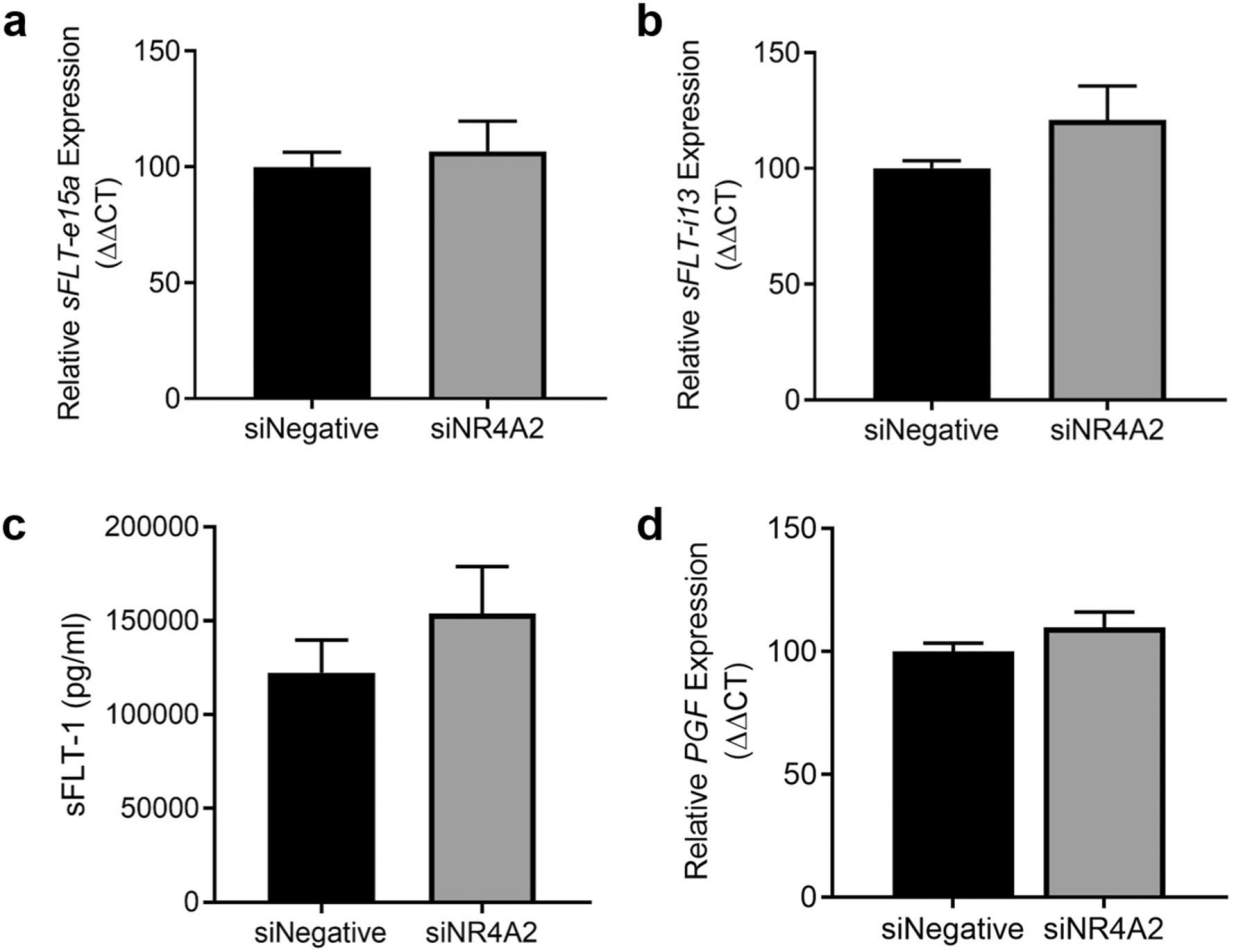
Primary cytotrophoblast expression and secretion of anti- and pro-angiogenic factors, sFLT-1 and PGF under hypoxic (1% O_2_) conditions. Expression assessed by qPCR and secretion by ELISA. There were no significant differences in expression of either sFLT-1 isoform mRNA expression, *sFLT-e15a* (**a**) or *sFLT-i13* (**b**), nor sFLT-1 protein secretion (**c**) or *PGF* mRNA expression (**d**) under hypoxia. Data presented as fold change from control ± SEM. n = 3 experimental replicates, with each sample from a different patient. Each experiment was run in triplicate.

We next assessed the effects of *NR4A2* knockdown on sFlt-1, an anti-angiogenic factor increased in preeclampsia, and placental growth factor (*PGF*), an angiogenic factor decreased in preeclampsia. Silencing *NR4A2* in cytotrophoblasts under hypoxia had no significant effect on the expression of the sFlt-1 isoforms, *sFlt-1*-*e15a* and *sFlt-1*-*i13* (Fig. 5a,b) or sFlt-1 secretion (Fig. 5c). Silencing *NR4A2* under hypoxia had no significant effect on *PGF* expression (Fig. 5d). Silencing *NR4A2* under normoxic conditions did not alter *sFlt-1* and *PGF* expression or sFlt-1 secretion (Supplementary Fig. S4).

### Silencing *NR4A2* in cytotrophoblasts under hypoxia alters expression of genes associated with oxidative stress, inflammation, and placental insufficiency

**Figure 6:**
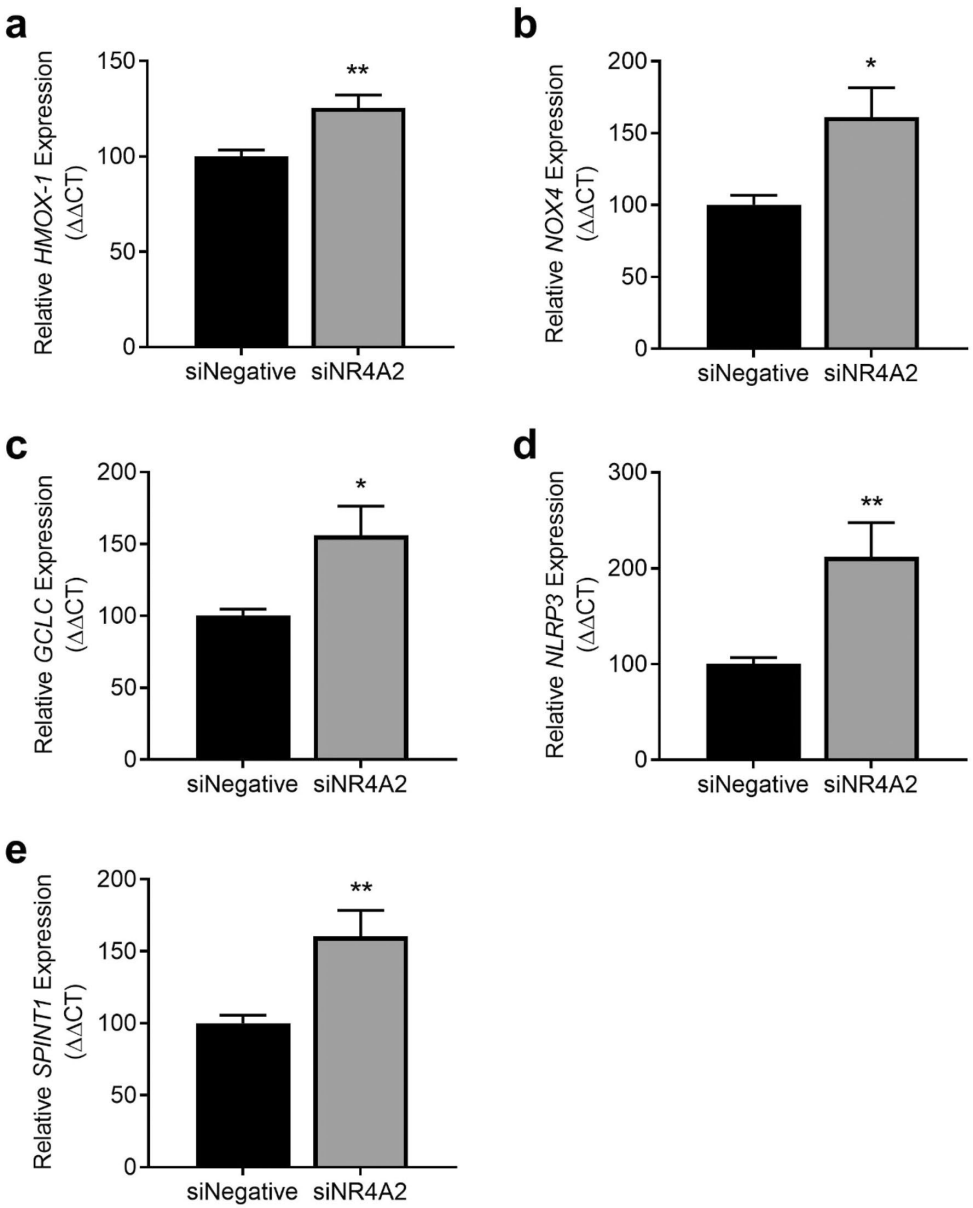
Effect of *NR4A2* knockdown in primary cytotrophoblasts on expression of genes associated with oxidative stress, inflammation, and placental insufficiency under hypoxic (1% O_2_) conditions. The expression of *HMOX-1* (**a**)*, NOX4* (**b**)*, GCLC* (**c**)*, NLRP3* (**d**) and *SPINT1* (**e**) were significantly increased with *NR4A2* knockdown compared to negative control. Data presented as relative change from control ± SEM. *p < 0.05, **p < 0.01. n = 3 experimental replicates, with each sample from a different patient. Each experiment was run in triplicate.

To identify pathways that *NR4A2* may be working through in the placenta, we assessed expression of genes associated with important placental cellular functions following *NR4A2* knockdown. Silencing *NR4A2* under hypoxia increased expression of genes involved in oxidative stress pathways including: haemoxygenase-1 (*HMOX-1*; *p* = 0.0036; Fig. 6a), NADPH oxidase 4 (*NOX4*; *p* = 0.0155; Fig. 6b) and Glutamate-Cysteine Ligase Catalytic Subunit (*GCLC*; *p* = 0.0162; Fig. 6c), but did not alter NAD(P)H Quinone Dehydrogenase 1 (*NQO1*; Supplementary Fig. S5e) or thioredoxin (*TXN*; Supplementary Fig. S5f.).

Knockdown of *NR4A2* under hypoxia significantly increased expression of NLR family pyrin domain containing 3 (*NLRP3*), a master regulator of the inflammasome (*p* = 0.0065; Fig. 6d) and Serine Peptidase Inhibitor, Kunitz Type 1 (SPINT1), associated with placental insufficiency (*p* = 0.0056; Fig. 6e).

Under hypoxia, silencing *NR4A2* did not alter expression of pro-apoptotic gene: BCL2 Associated X (*BAX;* Supplementary Fig. S5a), pro-survival gene: B-cell lymphoma 2 (*BCL2*; Supplementary Fig. S5b) or placental growth and proliferation genes: epidermal growth factor receptor (*EGFR*) and insulin-like growth factor 2 (*IGF2*) (Supplementary Fig. S5c and S5d).

Gene expression was also assessed in cytotrophoblasts with silenced *NR4A2* under normoxic conditions. In these cells we found significantly decreased expression of *BAX* (*p* = 0.0123; Supplementary Fig. S6a), *EGFR* (*p* = 0.0301; Supplementary Fig. S6c) and *IGF2* (*p* = 0.0178; Supplementary Fig. S6d). Under normoxic conditions, silencing NR4A2 did not alter levels of *BCL2*, *NOX4*, *HMOX-1*, *NQO1*, *TXN*, *GCLC, NLRP3*, or *SPINT1* (Supplementary Fig. S6b, e-k).

### *NR4A2* is not altered with TNF-α-induced endothelial dysfunction or pravastatin treatment

**Figure 7:**
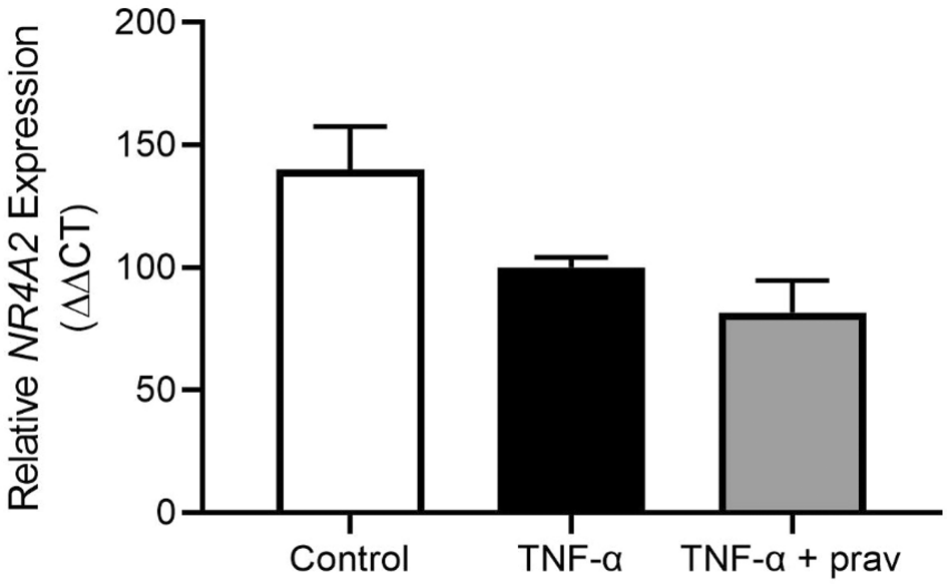
*NR4A2* expression in human umbilical vein endothelial cells with TNF-α induced dysfunction, and pravastatin treatment. There was no significant change in *NR4A2* mRNA expression with the addition of TNF-α. Pravastatin treatment did not alter *NR4A2* expression from TNF-α only levels. The control group was not exposed to TNF-α. Data presented as fold change from TNF-α treatment ± SEM. n = 3 experimental replicates, with each sample from a different patient. Each experiment was run in duplicate.

Given there was no change in *NR4A2* expression in the pathological preterm placenta, we assessed whether the increased circulating levels of *NR4A2* transcripts in pregnancies complicated by fetal growth restriction and preeclampsia might originate in the vasculature. We assessed this in endothelial cells, which form the inner lining of blood vessels. *NR4A2* expression was detectable in Human Umbilical Vein Endothelial cells (HUVECs). Moreover, as preeclampsia is associated with endothelial dysfunction^37^, we assessed whether dysfunction may alter *NR4A2* expression. Tumor necrosis factor (TNF)-α, an inflammatory mediator increased in the circulation of women with preeclampsia^38^, was used to induce endothelial dysfunction in HUVECs. Our model of endothelial dysfunction revealed that TNF-α-induced endothelial dysfunction did not alter *NR4A2* expression (Fig. 7). Furthermore, treatment with 200 µM pravastatin with TNF-α, a candidate drug for prevention of preeclampsia^39^, did not significantly alter *NR4A2* expression (Fig. 7).

## Discussion

Circulating nucleic acid transcripts involved in fetal-maternal signalling^40^ may provide a means for early identification of conditions involving placental dysfunction, such as preeclampsia and fetal growth restriction^12,13^. In this study, we extended the findings reported previously^13^, identifying that preeclampsia further increased circulating *NR4A2* transcript levels in pregnancies complicated by preterm fetal growth restriction (< 34 weeks gestation). However, this finding is not mirrored in placental tissue, where we observed no difference in *NR4A2* expression in placentas from cases of preterm growth restriction or preeclampsia (≤ 34 weeks gestation). We aimed to elucidate the role of *NR4A2* in the placenta, finding that *NR4A2* expression is higher in term placenta compared to preterm tissue, and its expression is significantly decreased in cytotrophoblast under hypoxia. Furthermore, we identified that decreased cytotrophoblast expression of *NR4A2* is associated with the dysregulation of genes involved in oxidative stress, inflammation, and growth and development.

A key aim of this paper was to identify whether expression of *NR4A2* in the pathological placenta mirrored the increased *NR4A2* transcripts demonstrated in the maternal circulation of cases of preterm preeclampsia and growth restriction (< 34 weeks gestation)—if so, supporting the concept that they may originate in the dysfunctional placenta. However, we did not find any significant change in *NR4A2* expression or protein in placentas from pregnancies complicated by preterm fetal growth restriction or preeclampsia. This contrasts the findings by Enquobahrie et al. who found a significant reduction in *NR4A2* expression in preeclamptic placental tissue compared to controls^41^. However, their study included both preterm and term samples. As we’ve shown that *NR4A2* expression is altered between preterm and term gestations, we suggest that this disparity is likely due to gestational differences. Further, early- and late-onset preeclampsia have distinct molecular processes, with the early-onset cases associated with increased severity^42,43^. Our study overcomes this potential confounder by assessing the harder to source clinical samples exclusively from cases of preterm preeclampsia delivering ≤ 34 weeks. Choosing early-onset cases also allows us to compare levels of placental mRNA to circulating transcripts in the FOX blood samples. Our findings suggest that the growth restricted or preeclamptic placenta is not the origin of the altered circulating mRNA transcripts. Furthermore, we identified that silencing *NR4A2* did not alter the expression or secretion of *sFlt-1* or *PGF,* key factors central to the pathogenesis of preeclampsia, suggesting that placental *NR4A2* is not involved in driving the release of anti-angiogenic factors from the dysfunctional placenta, a key process in the pathogenesis of preeclampsia. However, we have yet to distinguish whether placental *NR4A2* may have a role in late-onset preeclampsia, as we only assessed *NR4A2* levels in samples collected from early-onset cases of preeclampsia with preterm delivery (≤ 34 weeks).

Though *NR4A2* expression has been previously detected in the placenta^20,21,41^, alterations in placental expression across gestation had not yet been determined. We found that *NR4A2* expression was higher at term compared to preterm gestation. Upregulation in placental *NR4A2* has been hypothesised to be associated with inflammatory processes^21,44^. To further understand these changes across gestation, it would be beneficial to examine *NR4A2* in first trimester placental tissue, where, if *NR4A2* is involved in inflammation as hypothesised, there may be increased *NR4A2* expression given many inflammatory pathways are integral in driving successful extravllous cytotrophoblast invasion and critical arteriole remodelling^20^.

Although *NR4A2* was not significantly altered in the preterm pathological placenta, there is potential it could play a role in the pathophysiology underpinning placental dysfunction and disease. Our in vitro models simulate conditions that drive placental dysfunction, offering insight into whether *NR4A2* has a role in placental development, and the development of placental disease. Placental hypoxia is a feature of placental dysfunction, where impaired uteroplacental blood supply can cause periods of abnormally low oxygen tension^8^. Simulating these conditions, we found that hypoxia significantly reduced *NR4A2* expression in cytotrophoblast, but not protein. It is understood that changes in mRNA expression do not always translate to the same changes in protein production. In contrast, the placental explant tissue (presenting whole tissue expression) did not have altered *NR4A2* expression under hypoxia. This might be due to the heterogenous contribution and interactions of the multiple cell types in the placenta besides trophoblast cells, including vascular, immune and stromal cells.

This study identified several potential actions that *NR4A2* expression may regulate in the placenta specific cytotrophoblast cells under dysfunctional conditions. Silencing *NR4A2* under hypoxia increased expression of the cytoprotective antioxidant genes *HMOX-1* and *GCLC*. Indeed, given placental dysfunction is associated with oxidative stress^45^, this suggested that reducing *NR4A2* expression could be beneficial. Intriguingly, silencing *NR4A2* under hypoxia also increased the expression of *SPINT1*. Our team has recently reported the important finding that low expression of *SPINT1* in the human placenta and maternal circulation is associated with placental insufficiency and growth restriction^9^. Again, this result suggests that therapeutic interventions that reduce *NR4A2* may be advantageous.

However, we also found that silencing *NR4A2* increased the expression of *NLRP3*, which is a key regulator of the inflammatory response and has been implicated in preeclampsia pathogenesis^46^. *NR4A2* has been previously identified as a *NLRP3* inflammasome activation-responsive gene in a human monocyte cell line, with the suggestion that its induction acts in a negative feedback loop to prevent sustained inflammasome activation^47^. This implies, in contrast to our first suggestion, that loss of *NR4A2* may result in persistent inflammation, detrimental to the placenta. Furthermore, silencing *NR4A2* also increased expression of *NOX4*, a marker of oxidative stress. Enhancement of oxidative stress could be harmful to the already stressed placenta. Thus, these findings reveal that a more complex regulation may be at play and further studies are required to gain a better understanding of compensation and causation.

As *NR4A2* acts as a transcription factor^48^, it was not surprising that it was involved in the regulation of many different pathways in cytotrophoblast cells. These results have allowed us to identify the pathways associated with *NR4A2*, but the conflicting responses mean we cannot clearly conclude whether reduction of *NR4A2* in cytotrophoblast cells is a harmful consequence of hypoxic stress or a beneficial adaptation to mediate hypoxic damage in the placenta. Advanced protein assessment could be undertaken in the future to validate these findings.

Although this work predominantly aimed to assess *NR4A2* expression and function in the dysfunctional placenta, we were also able to look at the function of *NR4A2* at a physiologically normal oxygen tension. We identified several important genes to be differentially expressed in normoxic conditions with *NR4A2* suppression, not altered under hypoxia. Silencing *NR4A2* beneficially decreased cytotrophoblast levels of *BAX*, a pro-apoptotic gene, but also adversely decreased expression *IGF2* and *EGFR*, which are markers of cell proliferation and growth. These findings indicate that *NR4A2* responds variably under different oxygen tensions. However, once again it is unclear whether reduction of *NR4A2* expression may be beneficial.

Given these results suggest that the placenta is unlikely to be the source of increased circulating *NR4A2* transcripts in individuals with pregnancies complicated by fetal growth restriction and preeclampsia, we examined whether the vasculature may be the source. Though we could detect *NR4A2* expression in endothelial cells, we did not find increased expression under TNF-α-induced dysfunction. This suggests that the endothelium is also unlikely to be the origin of the increased circulating *NR4A2* transcripts—but additional studies using additional endothelial cell types beyond HUVECs, and different inducers of endothelial dysfunction are required before this can be confirmed. An important consideration is that the circulating transcripts may also originate from other organs in the maternal system that we did not assess here. Additionally, treatment with the therapeutic pravastatin, a drug currently in trial to prevent preeclampsia, had no effect on *NR4A2* expression under endothelial dysfunction, suggesting that pravastatin treatment does not affect regulation of this transcription factor.

## Conclusion

The origin of elevated circulating *NR4A2* transcripts in patients with preterm fetal growth restriction and preeclampsia remains unknown, but is unlikely to be from the placenta. Although we found that placental *NR4A2* expression is not altered with preterm preeclampsia or fetal growth restriction, the gene does regulate several important intracellular pathways associated with oxidative stress, fetal growth, and inflammation under the dysfunctional condition of hypoxia. More research is required to confirm the role of *NR4A2* in the placenta, and whether altering the expression and localisation of *NR4A2* levels may provide a pathway to enhancing the regulation of protective pathways in placental dysfunction.

## Methods

### Fetal oxygenation (FOX) study

Maternal blood was collected from women whose pregnancies were complicated by preterm fetal growth restriction across six tertiary hospitals in Australia and New Zealand, as previously described^13^. The blood was directly collected (after corticosteroid administration, prior to delivery) into PAXgene^®^ Blood RNA tubes (Pre-Analytix, Hombrechtikon, Switzerland) and processed according to manufacturer’s instructions.

Ethical approval was obtained from human research ethics committees at all institutions (Mercy Health Human Research Ethics Committee R11/04, Royal Women’s Hospital + Sunshine Hospital: Royal Women’s Hospital Human Research Ethics Committee 10/41, Mater Mothers’ Hospital: Mater Human Research Ethics Committee 1928 M, Royal Hospital for Women: South Eastern Sydney Local Health District Human Research Ethics Committee 12/240, Royal North Shore Hospital: Northern Sydney Local Health District Human Research Ethics Committee 1305-151 M, National Women’s Health, Auckland City Hospital: Health and Disability Ethics Committee 12/NTA/96) and all women provided informed, written consent. Experiments were performed following the relevant institutional guidelines and regulations.

Preterm fetal growth restriction was defined as a birthweight < 10th centile (www.gestation.net, Australian parameters) requiring iatrogenic delivery prior to 34 weeks’ gestation with uteroplacental insufficiency (asymmetrical growth + abnormal artery Doppler velocimetry +/− oligohydramnios +/− abnormal fetal vessel velocimetry). Fetal growth restriction due to infection, chromosomal or congenital abnormalities, and multiple pregnancy was excluded. For our sub-analysis in the current study, the cases of growth restriction were split into preeclamptic and normotensive groups. Patient clinical characteristics are presented in Table 1.

**Table 1:** Patient characteristics of pregnancies complicated with fetal growth restriction with and without preeclampsia.

Characteristics	Normotensive (n = 45)	Preeclampsia (n = 71)
Maternal age, years	32 (27.5–34.5)	33 (30–37)
Nulliparity	24 (53%)	45 (63%)
Body-mass index, kg/m^2^	24 (21–26.3)	27 (22–32) *
Smoking during pregnancy	11 (24%)	4 (6%)
Diabetes during pregnancy	8 (17%)	8 (11%)
Absent or reversed end diastolic flow in umbilical artery	19 (42%)	28 (40%)
Gestational age at delivery, weeks	31.4 (30.1–32.45)	30 (28.3–32) **
Birthweight, grams	1090 (811.5–1304)	1094 (788–1300)
Estimated fetal weight, grams	1009 (746–1264)	1044 (713.8–1304)
Male sex	27 (60%)	42 (59%)
Umbilical artery pH, median	7.28 (7.25–7.32)	7.27 (7.22–7.3)
Umbilical artery pH < 7.2	6 (13%)	8 (11%)
Neonatal deaths within 42 days of birth	0 (0%)	2 (3%)

Data are n (%),or median (IQR). Missing BMI data for n = 3 normotensive samples and n = 6 preeclamptic samples, and umbilical artery pH for n = 1 preeclamptic sample. Estimated fetal weight not available for n = 4 normotensive and n = 5 preeclamptic samples. *p < 0.05, **p < 0.01.

### Placenta and umbilical cord collection

Ethical approval was obtained from the Mercy Health Human Research Ethics Committee (R11/34). Women presenting to the Mercy Hospital for Women (Heidelberg, Victoria) gave informed, written consent for the collection of their placenta and umbilical cord. Experiments were performed following institutional guidelines and regulations.

Placentas were obtained from pregnancies complicated by early-onset preeclampsia (requiring delivery ≤ 34 weeks’ gestation). Preeclampsia was defined according to the American College of Obstetricians and Gynecologists guidelines published in 2013^49^. Placentas were also obtained from cases of preterm fetal growth restriction (requiring delivery ≤ 34 weeks gestation) defined as a birthweight < 10th centile, according to Australian population charts^50^. Placental tissue from cases associated with congenital infection, chromosomal or congenital abnormalities and multiple pregnancies were excluded.

Term (delivery 37–41 weeks’ gestation) and preterm placentas (delivery 24–36 weeks’ gestation) were also collected from normotensive pregnancies where a fetus of normal birthweight percentile (> 10th centile relative to gestation), and no clinical evidence of growth restriction, was delivered. Preterm deliveries in this group were predominantly for iatrogenic reasons (including vasa/placenta previa and suspected placental abruption) or premature rupture of membranes. Cases with hypertensive disease or evidence of chorioamnionitis (confirmed by placental histopathology) were excluded.

Placental tissue was collected within 30 min of delivery. For the groups detailed above, tissue was cut from four sites of the placenta and washed in cold phosphate buffered saline (PBS; 137 mM NaCl, 10 mM Na_2_HPO_4_, 1.8 mM KH_2_PO_4_, 2.7 mM KCl, pH 7.4) before being placed in RNAlater™ for 48 h. The tissue was then snap frozen and stored at − 80 °C for subsequent analysis. Patient characteristics are presented in Tables 2, 3 and 4.

**Table 2:** Patient characteristics for placental tissue used to assess *NR4A2* expression between preterm and term gestation.

	Preterm (n = 30)	Term (n = 29)
**Maternal age, years** Median (IQR)	32 (28–36)	31.5 (29.5–36.5)
**Gestational age at sample collection, weeks** Median (IQR)	33.75 (30.53–39.1)	39.1 (38.85–40.25) ****
**Maternal Body Mass Index (kg/m**^**2**^**)** Median (IQR)	25.1 (22.2–28.85)	24.5 (22.4–27.70)
**Parity**		
**0**	10 (33%)	10 (34%)
**1**	12 (40%)	16 (55%)
** ≥ 2**	8 (27%)	3 (10%)
**Mode of Delivery**		
**Vaginal (%)**	0 (0)	0 (0)
**Caesarean Section (%)**	30 (100%)	29 (100%)
**Birthweight (g)** Median (IQR)	2074 (1518–2701)	3480 (3150–3770) ****

Maternal age unavailable for n = 1 term sample. BMI data unavailable for n = 6 preterm samples. ****p < 0.0001.

**Table 3:** Patient characteristics of cases with preeclampsia, fetal growth restriction and control samples for gene (mRNA) expression studies.

	Preterm controls (n = 10)	Preeclampsia (n = 49)	Fetal growth restriction (n = 14)
**Maternal age, years** Median (IQR)	34 (26.5–37.5)	31 (27–35.5)	30 (25.3–33.5)
**Gestational age at delivery, weeks** Median (IQR)	30 (29.4–31.6)	30 (27.8–31.3)	32.7 (30.9–34.0)*
**Maternal Body Mass Index (kg/m**^**2**^**)** Median (IQR)	28.4 (24.0–30.0)	27 (23.9–36.05)	25.8 (18.75–29.5)
**Parity no. (%)**			
**0**	2 (20.0)	35 (71.4)	9 (64.3)
**1**	4 (40.0)	9 (18.4)	2 (14.3)
** ≥ 2**	4 (40.0)	5 (10.2)	3 (21.4)
**Highest SBP prior to delivery (mmHg)** Median (IQR)	120 (110–126.3)	175 (160–184.5)****	120 (115–126.3)
**Highest DBP prior to delivery (mmHg)** Median (IQR)	70 (67.5–76.25)	100 (97.5–110)****	76.5 (70–83.5)*
**Mode of Delivery**			
**Vaginal (%)**	0 (0)	0 (0)	0 (0)
**Caesarean Section (%)**	10 (100)	(0)	14 (100)
**Birthweight (g)** Median (IQR)	1496 (1322–2011)	1087 (843.5–1421)**	1182 (973–1658)
**Male sex (%)**	4 (40)	29 (59)	7 (50)

BMI data unavailable for n = 3 preterm controls, n = 1 fetal growth restriction samples. Birthweight data unavailable for n = 1 preeclamptic sample. Statistical analysis compared the preeclamptic or fetal growth restricted samples to the preterm controls. *p < 0.05, **p < 0.01, ****p < 0.0001.

**Table 4:** Patient characteristics of women with preeclampsia, fetal growth restriction and preterm control samples for protein studies.

	Preterm controls (n = 15)	Preeclampsia (n = 31)	Fetal growth restriction (n = 17)
**Maternal age, years** Median (IQR)	30 (21–37)	29 (26–33)	29 (26.5–34)
**Gestational age at delivery, weeks** Median (IQR)	30 (27.6–31.4)	29.7 (27.9–31.9)	32.3 (31.2–34) ***
**Maternal Body Mass Index (kg/m**^**2**^**)** Median (IQR)	28 (24–35.3)	28.3 (25–37.2)	25 (19.2–29.8)
**Parity no. (%)**			
**0**	4 (26.7)	22 (71.97)	11 (64.7)
**1**	8 (53.3)	6 (19.35)	2 (11.8)
** ≥ 2**	3 (20)	3 (9.68)	4 (23.5)
**Highest SBP prior to delivery (mmHg)** Median (IQR)	120 (110–130)	175 (170–184)****	120 (115–127)
**Highest DBP prior to delivery (mmHg)** Median (IQR)	70 (63–80)	100 (90–110)****	75 (70–84)*
**Mode of delivery (%)**			
**Vaginal**	0 (0)	0 (0)	0 (0)
**Caesarean Section**	15 (100)	31 (100)	17 (100)
**Birthweight (g)** Median (IQR)	1451 (971–1790)	1061 (770–1407)*	1126 (958–1632)
**Male sex (%)**	6 (40)	17 (55)	10 (59)

BMI data unavailable for n = 4 preterm control, n = 7 preeclamptic, n = 1 fetal growth restricted samples. *p < 0.05, ***p < 0.001, ****p < 0.0001. Statistical analysis was by *t* test comparing the preeclamptic or fetal growth restricted samples to the preterm controls.

Placentas were also obtained from healthy, normotensive term pregnancies (≥ 37 weeks’ gestation) at elective caesarean section for explant dissection and cytotrophoblast isolation. Umbilical cords were collected for the isolation of Human Umbilical Vein Endothelial Cells (HUVECs).

### Collection and culture of placental explants and hypoxia treatment

Placental explants were collected with maternal and fetal surfaces removed by careful dissection. Three placental pieces per well were cultured in 24-well plates (10–15 mg per well), in Gibco™ Dulbecco's Modified Eagle Medium (DMEM; ThermoFisher Scientific, Scoresby, Vic) supplemented with 10% fetal calf serum (FCS; Sigma-Aldrich, St Louis, USA) and 1% Anti-Anti (AA; Life Technologies, Carlsbad, California, USA). Explants were cultured under 8% O_2_, 5% CO_2_ at 37 °C overnight (16–18 h). After replacement with fresh media (DMEM/10% FCS/1%AA), explant tissue was cultured at 37 °C, 5% CO_2_ for 48 h under 8% O_2_ (normoxic conditions) or 1% O_2_ (hypoxic conditions). This model demonstrates increased sFlt-1 expression and protein^51,52^, and induced HIF1α protein^53^ with hypoxia. Following this, explant tissue was snap frozen, and stored at -80 °C for subsequent analysis.

### Primary cytotrophoblast isolation and hypoxia treatment

Human primary cytotrophoblast were isolated from healthy, term placentas from elective caesarean section as previously described^54^. The cells were plated in media (DMEM/10% FCS/1%AA) on fibronectin (10 ug/mL; BD Bioscience, USA) coated culture plates. Viable cells were incubated at 37 °C, 8% O_2,_ 5% CO_2_ overnight to equilibrate and allow adhesion to cell culture plate. After replacement with fresh media (DMEM/10% FCS/1%AA), cells were incubated under either 8% O_2_ (normoxic conditions) or 1% O_2_ (hypoxic conditions) at 37 °C, 5% CO_2_. After 48 h, the cells were collected for RNA and protein extraction.

### Silencing of *NR4A2* in primary human cytotrophoblasts

Pre-designed short interfering RNAs (siRNAs) against *NR4A2* (M-003427-02-0005; Dharmacon, Lafayette, California, USA) or a pre-tested negative siRNA (Qiagen, Valencia, CA, USA) were combined with lipofectamine (RNAiMax; Invitrogen) in Optimem media (ThermoFisher Scientific) and allowed to complex for 20 min at room temperature. After equilibration of isolated cytotrophoblasts overnight, fresh trophoblast media (DMEM/10% FCS, no AA) was added to each well and 10 nM siRNA solutions added in a dropwise manner. The cells were incubated in either 8% O_2_ (normoxic conditions) or 1% O_2_ (hypoxic conditions) at 5% CO_2_ for 48 h. Media and cell lysates were collected for subsequent analysis.

### MTS cell viability assay

Cell viability was assessed after siRNA treatment using an MTS assay. CellTiter 96-AQueous One Solution (Promega, Madison WI) was used according to the manufacturer’s instructions. Optical density was measured using a Bio-Rad X-Mark Microplate Spectrophotometer (Hercules, CA, USA) and Bio-Rad Microplate Manager 6 software.

### Endothelial cell isolation and culture

HUVECs were isolated from the umbilical cord of normotensive pregnancies as previously described^55^. The HUVECs were cultured in M199 media (Life Technologies, California, USA) containing 20% newborn calf serum, 1% endothelial cell growth factor, 1% heparin (Sigma-Aldrich) and 1% AA and used between passages 1–3. Cells were plated in 24-well plates containing M199 media supplemented with 10% FCS, 1% endothelial cell growth factor, 1% heparin and 1% AA.

### Endothelial dysfunction and statin treatment

To induce endothelial dysfunction, the HUVECs were pre-treated with 10 ng/mL tumour necrosis factor (TNF)-α, and incubated at 37 °C, 20% O_2_ and 5% CO_2_ for 2 h as previous^53,56^. Following this, 200 µM pravastatin (candidate drug for the treatment of preeclampsia) (Sigma-Aldrich) was added and cells incubated for 24 h. Cell lysates were collected for subsequent analysis.

### Real time polymerase chain reaction (RT-PCR)

Total RNA was extracted from whole blood using the PAXgene^®^ Blood miRNA Kit (Pre-Analytix) as described previously^13^. RNA was extracted from placental tissue, placental explants, cytotrophoblast, and HUVECs using the Qiagen RNeasy Mini Kit following manufacturers’ instructions. The RNA was quantified using a Nanodrop 2000 spectrophotometer (ThermoFisher Scientific, Waltham, MA, USA). Extracted RNA was converted to cDNA using the Applied BiosystemsTM High-Capacity cDNA Reverse Transcription Kit, following manufacturer guidelines on the iCycler iQ5 (Bio-Rad). Quantitative Taqman PCR (Life Technologies) was performed to quantify mRNA expression of *NR4A2* (Hs00428691_m1)*, PGF* (Hs00182176_m1)*, HMOX-1* (Hs01110250_m1), *NOX4* (Hs00418356_m1), *GCLC* (Hs00155249_m1), *NLRP3* (Hs00918082_m1), *SPINT1* (Hs00173678_m1), *BAX* (Hs00180269_m1), *BCL2* (Hs00608023_m1), *EGFR* (Hs01076078_m1), *IGF2* (Hs04188276_m1), *NQO1* (Hs00168547_m1) and *TXN* (Hs00828652_m1). Stability of reference genes was confirmed for each sample type and used appropriately; for blood *YWHAZ* (Hs01122454_m1), *B2M* (Hs00187842_m1) and *GUSB* (Hs00939627_m1) for cells *YWHAZ* (Hs01122454_m1) and for explants and placental tissue: *TOP1* (Hs00243257_m1) and *CYC1* (Hs00357717_m1). Taqman RT-PCR was performed on the CFX384 (BioRad) with the following run conditions: 50 °C for 2 min; 95 °C for 10 min, 95 °C for 15 s, 60 °C for 1 min (40 cycles) or 95 °C for 20 s; 95 °C for 3 s, 60 °C for 30 s (40 cycles; Taqman Fast Advanced Master Mix).

The sFlt-1 splice variants *sFlt-1-*i13 and *sFlt-1-*e15a were measured in a SYBR PCR with SYBR Green Master mix (Applied Biosystems) using primers specific for each variant. The primers for *i13* were 5′-ACAATCAGAGGTGAGCACTGCAA-3′ (forward) 5′-TCCGAGCCTGAAAGTTAGCAA-3′ (reverse), for *e15a* 5-CTCCTGCGAAACCTCAGTG-3′ (forward) 5′-GACGATGGTGACGTTGATGT-3′ (reverse) and for *YWHAZ* (reference gene) 5′-GAGTCATACAAAGACAGCACGCTA-3′ (forward) 5′-TTCGTCTCCTTGGGTATCCGATGT-3′ (reverse). The SYBR PCR was run on the CFX384 (Bio-Rad), with 40 cycles of 95 °C for 21 s, then 60 °C for 20 min. All data were normalized to the appropriate reference gene as an internal control and calibrated against the average Ct of the control samples. All cDNA samples were run in duplicate.

### Western blot analysis

Protein lysates were collected from human primary cytotrophoblasts and placental tissue collected from pregnancies complicated by preeclampsia, fetal growth restriction and preterm controls (≤ 34 weeks) using RIPA lysis buffer containing proteinase and phosphatase inhibitors (Sigma Aldrich). Protein concentration was assessed with Pierce™ BCA Protein Assay Kit (ThermoFisher Scientific, Massachusetts, USA). Placental lysates (20 µg) were separated on 4–20% Mini-PROTEAN® TGX™ Precast Protein Gels (Bio-Rad) and PVDF membranes (Millipore; Billerica, MA, United States). The membranes were blocked with 1% bovine serum albumin (BSA; Sigma-Aldrich), prior to overnight incubation with the primary antibody, (diluted 1:500 in 1%BSA/TBS-T; GTX133225, Sapphire Bioscience, NSW, Australia). Blots were incubated with secondary anti-rabbit antibody (W401, Promega, Madison WI, USA) at 1:2500 dilution in 5% skim milk for 1 h. Bands were visualized using a chemiluminescence detection system (GE Healthcare Life Sciences) and ChemiDoc XRS (Bio-Rad). β-actin acted as the loading control, (diluted 1:20,000 in 5% skim milk; Santa Cruz, Texas, USA). Relative densitometry was measured using Image Lab software (Bio-Rad).

### Enzyme linked immunosorbent assay (ELISA)

Soluble fms-like tyrosine kinase-1 (sFlt-1) secretion was measured in cytotrophoblast conditioned culture media using the DuoSet Human VEGF R1/FLT-1 kit (R&D systems by Bioscience, Waterloo, Australia) according to manufacturer’s instructions. Optical density was measured using a Bio-Rad X-Mark microplate spectrophotometer and Bio-Rad Microplate Manager 6 software.

### Statistical analysis

All in vitro experiments were performed with technical duplicates or triplicates and repeated with a minimum of three different patient samples. Data were tested for normal distribution and statistically tested as appropriate. Either an unpaired *t* test or Mann–Whitney test was used. All data are expressed as mean ± SEM. *P* values < 0.05 were considered significant. Statistical analysis was performed using GraphPad Prism software 8 (GraphPad Software, Inc.; San Diego, CA, USA).

## Supplementary Information

Supplementary Information.

## Data Availability

The datasets generated during and analysed during the current study are available from the corresponding author on reasonable request.
