# Supplementary material for: NR4A2 expression is not altered in placentas from cases of growth restriction or preeclampsia, but is reduced in hypoxic cytotrophoblast
Source: Sci Rep. 2021 Oct 19;11:20670. doi: 10.1038/s41598-021-00192-y (PMC8526588; doi:10.1038/s41598-021-00192-y)
Supplement: Supplementary file 1 — Supplementary Information. [file 41598_2021_192_MOESM1_ESM.docx]

**Supplementary File**

**NR4A2 expression is not altered in placentas from cases of growth restriction or preeclampsia, but is reduced in hypoxic cytotrophoblast**

Authors: Natasha de Alwis, Sally Beard, Natalie K. Binder, Natasha Pritchard, Tu’uhevaha J. Kaitu’u-Lino, Susan P. Walker, Owen Stock, Katie M. Groom, Scott Petersen, Amanda Henry, Joanne M. Said, Sean Seeho, Stefan C. Kane, Stephen Tong, and Natalie J. Hannan*

***Corresponding author:**

A/Prof Natalie Hannan; Department of Obstetrics and Gynaecology, University of Melbourne. Mercy Hospital for Women, 163 Studley Rd., Heidelberg 3084, Victoria, AUSTRALIA.

Ph: +613 8458 4371

Fax: +613 8458 4380

Email: nhannan@unimelb.edu.au

**
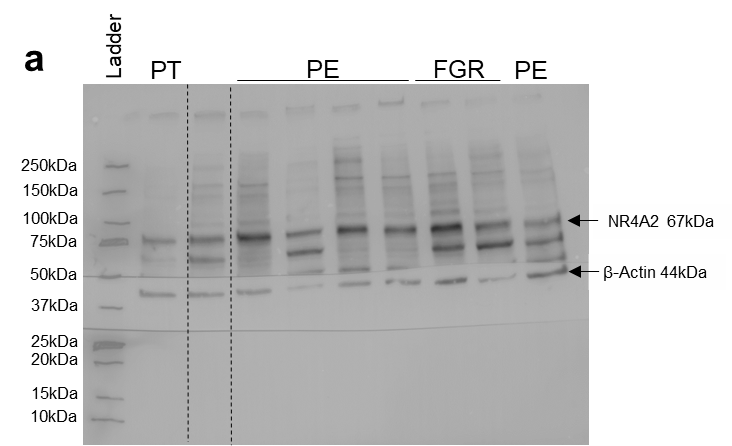

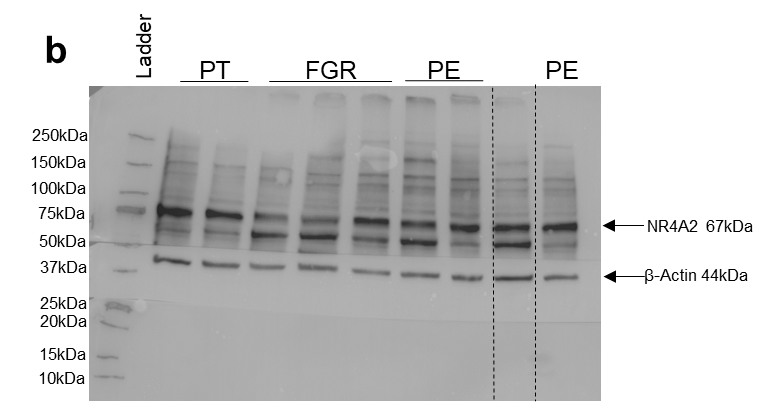

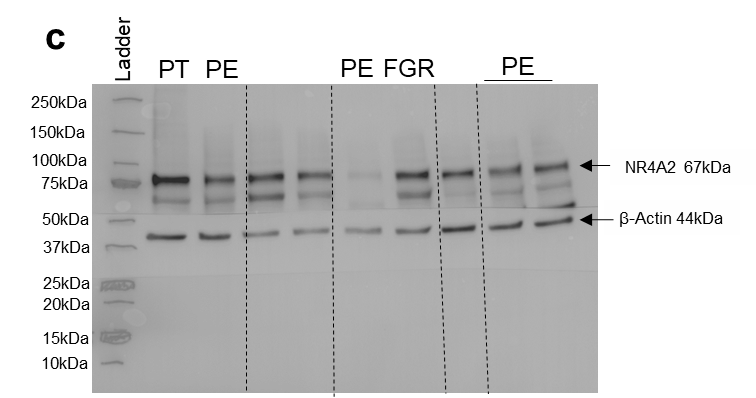
**

**
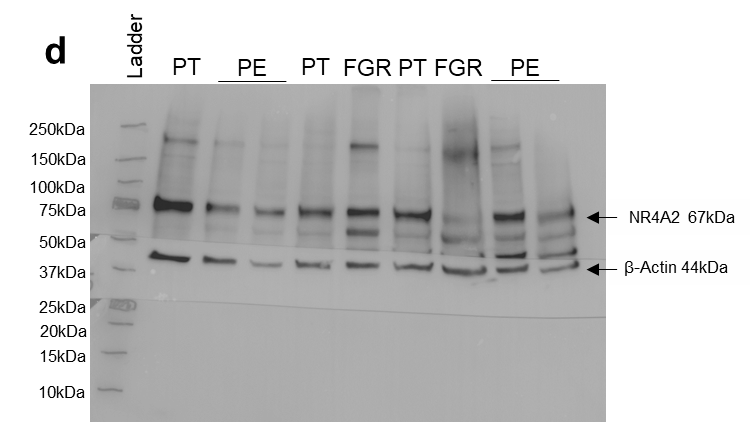

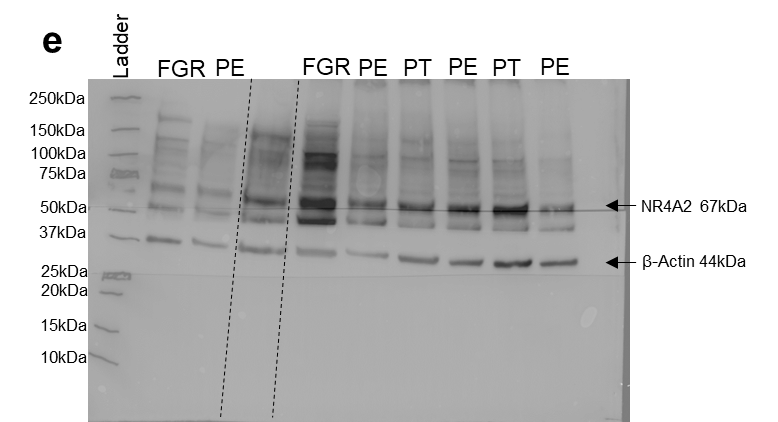

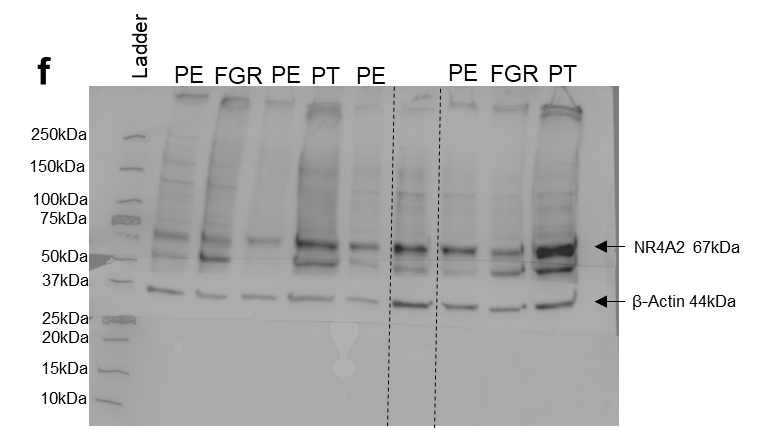
**

**
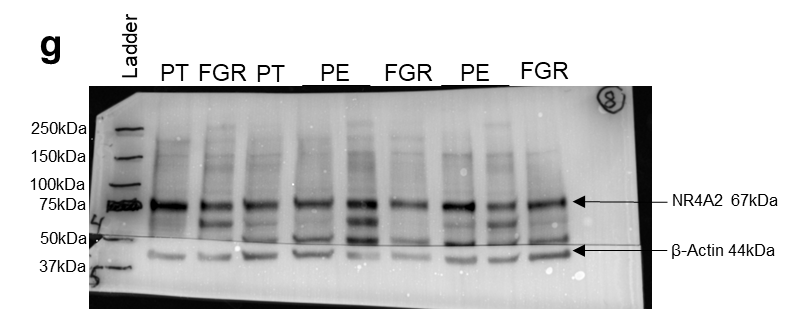

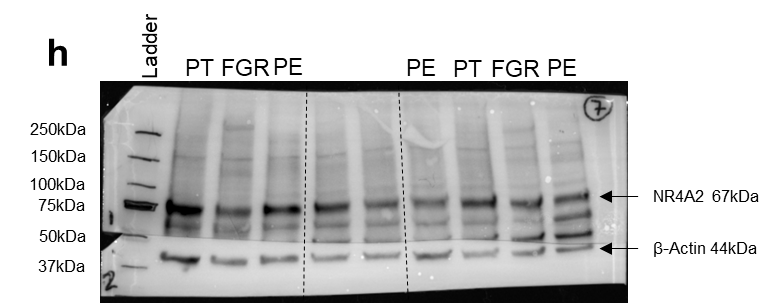
**

**Supplementary Fig. S1 Western blot images of NR4A2 protein in placental tissue collected from pregnancies complicated by preterm fetal growth restriction (FGR), preeclampsia (PE), and preterm controls (PT).** These blots (a-h) were used to derive the densitometric data presented in Fig. 3c and used to create a cropped image for Fig. 3b. Bands of interest are marked with arrows (β-actin acted as loading control). Samples that were not relevant to this study are demarcated with dashed lines, and were excluded from densitometric analysis. The labelled lanes were used in analysis; FGR n=17, PE n=31, PT n=15.


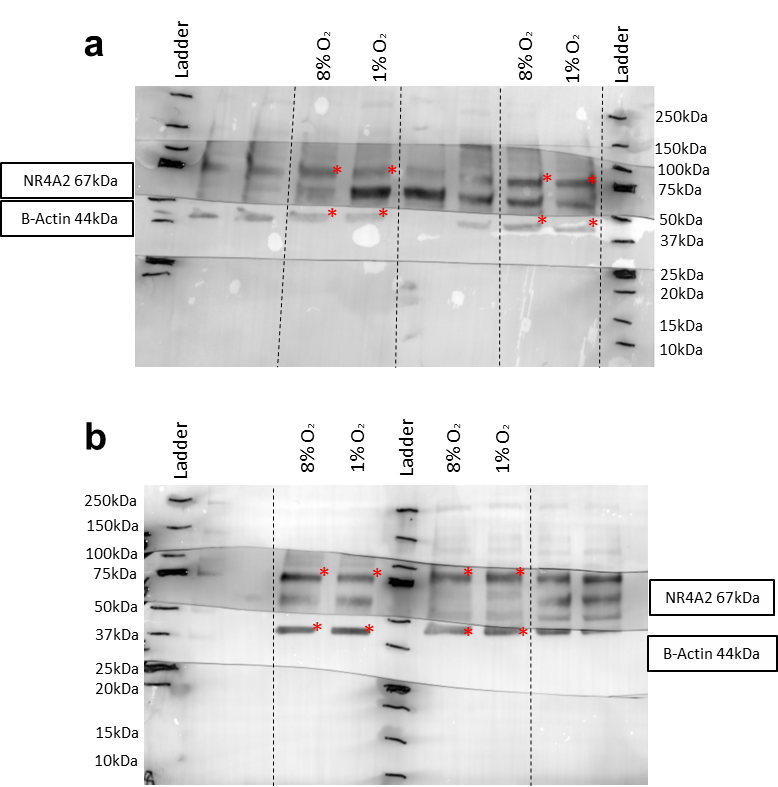


**Supplementary Fig. S2 Western blot images of NR4A2 protein in placental tissue under hypoxic (1% O_2_) and normoxic (8% O_2_; control) conditions.** These blots (a-b) were used to derive the densitometric data and cropped image presented in Fig.4c. Bands of interest are marked with an asterisk (*). β-actin acted as loading control. Samples that were not relevant to this study are demarcated with dashed lines, and were excluded from densitometric analysis. The labelled lanes were used in analysis; n=4.

**
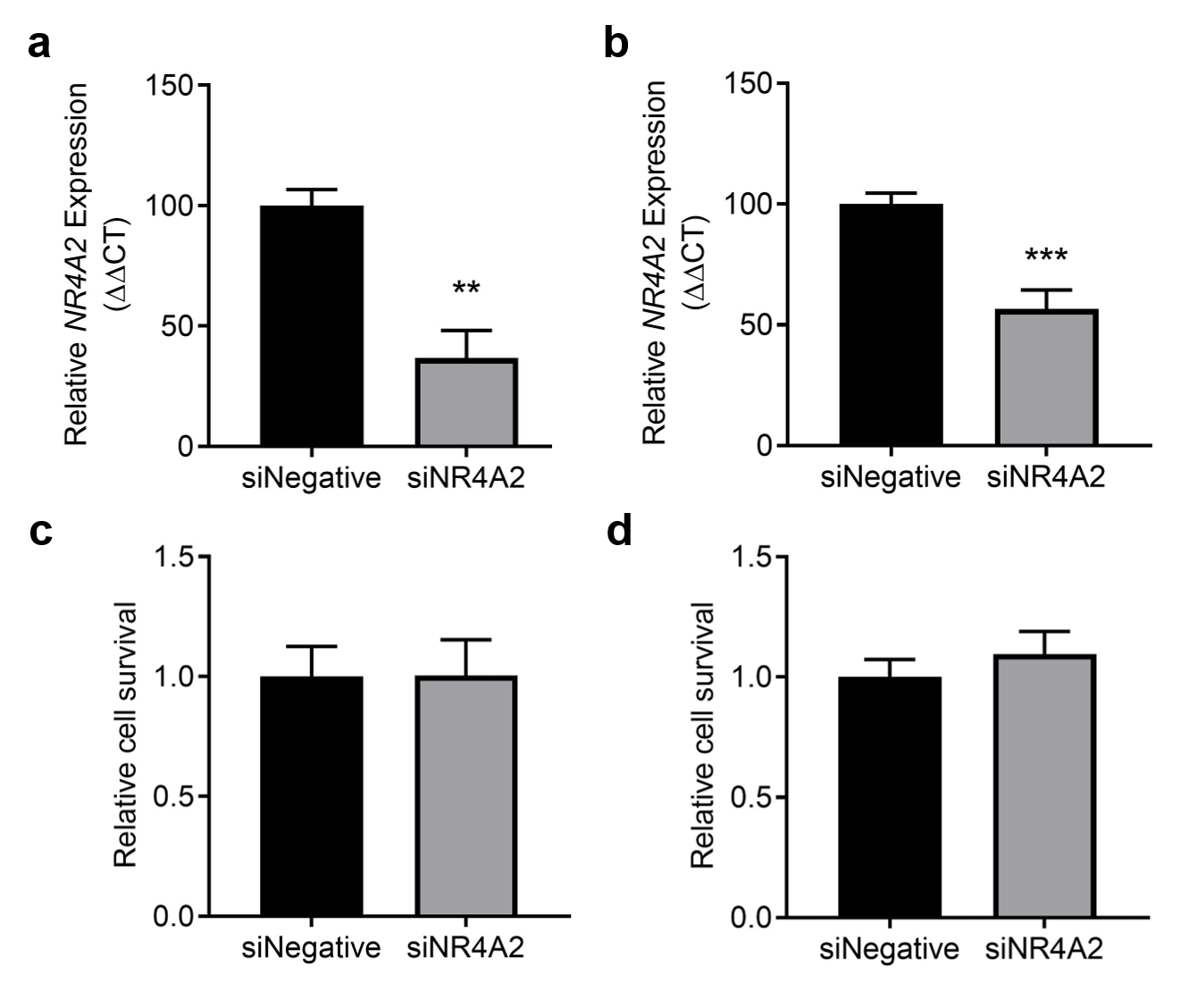
**

**Supplementary Fig. S3** *NR4A2* expression in cytotrophoblasts and cell survival with addition of silencing siRNAs under normoxic (8% O_2_; a, c) and hypoxic (1% O_2_; b, d) conditions. Expression assessed by qPCR (A, B) and cell survival by MTS Assay (C, D). *NR4A2* expression was significantly decreased under both oxygen tensions, and cell survival was unaltered. Data presented as relative change from control ± SEM. **p<0.01, ***p<0.001. n=3 experimental replicates, with each sample from a different patient. Each experiment was run in triplicate.


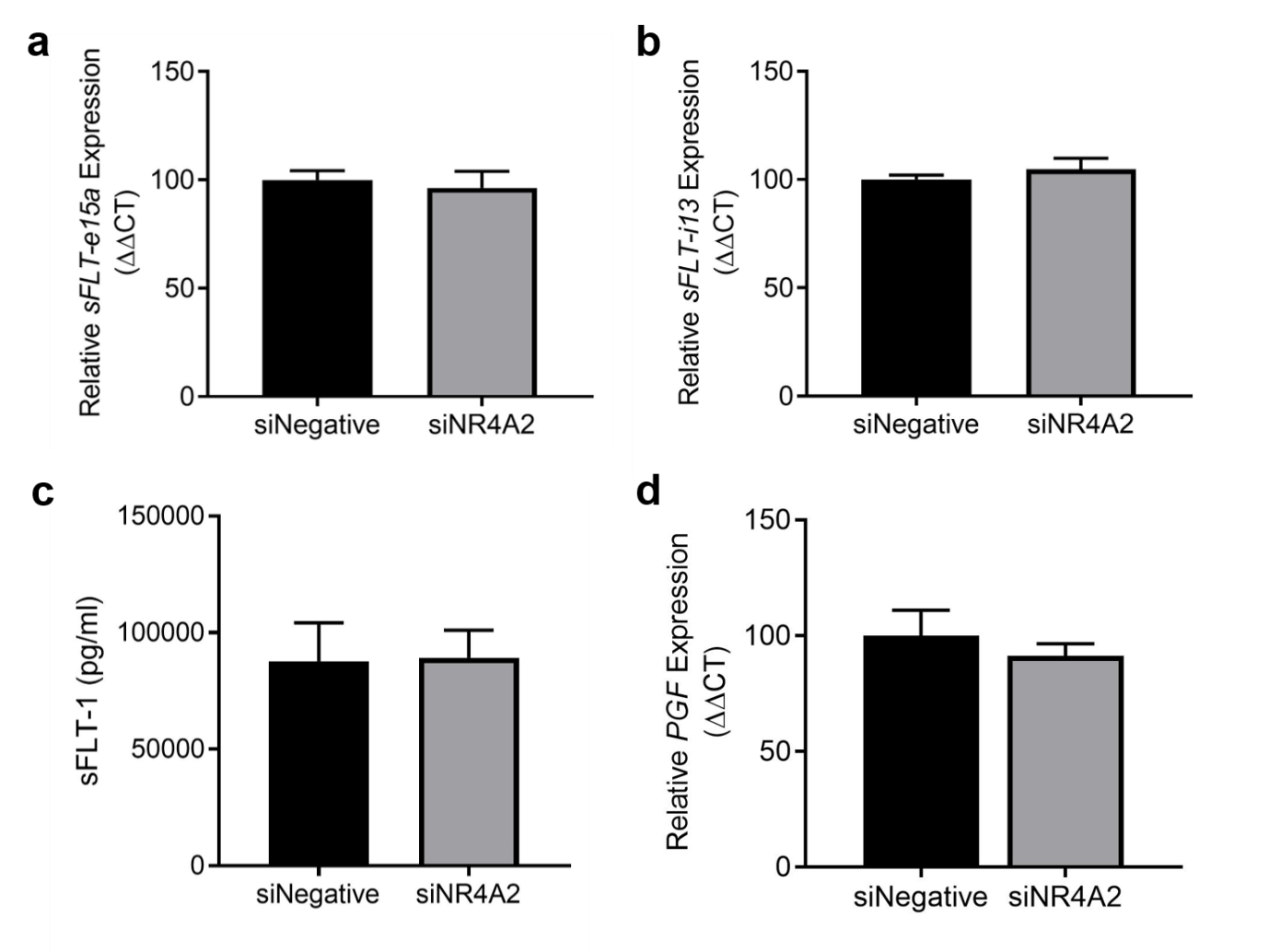


**Supplementary Fig. S4** Primary cytotrophoblast expression and secretion of anti- and pro-angiogenic factors, sFLT-1 and PGF under normoxic (8% O_2_) conditions. a) *sFLT-e15a* expression, b) *sFLT-i13* expression, c) sFLT-1 protein secretion, d) *PGF* expression. Expression assessed by qPCR and protein secretion by ELISA. There were no significant differences in expression in either isoform, nor sFLT-1 secretion under normoxic conditions. Data presented as relative change from control ± SEM. n=3 experimental replicates, with each sample from a different patient. Each experiment was run in triplicate.


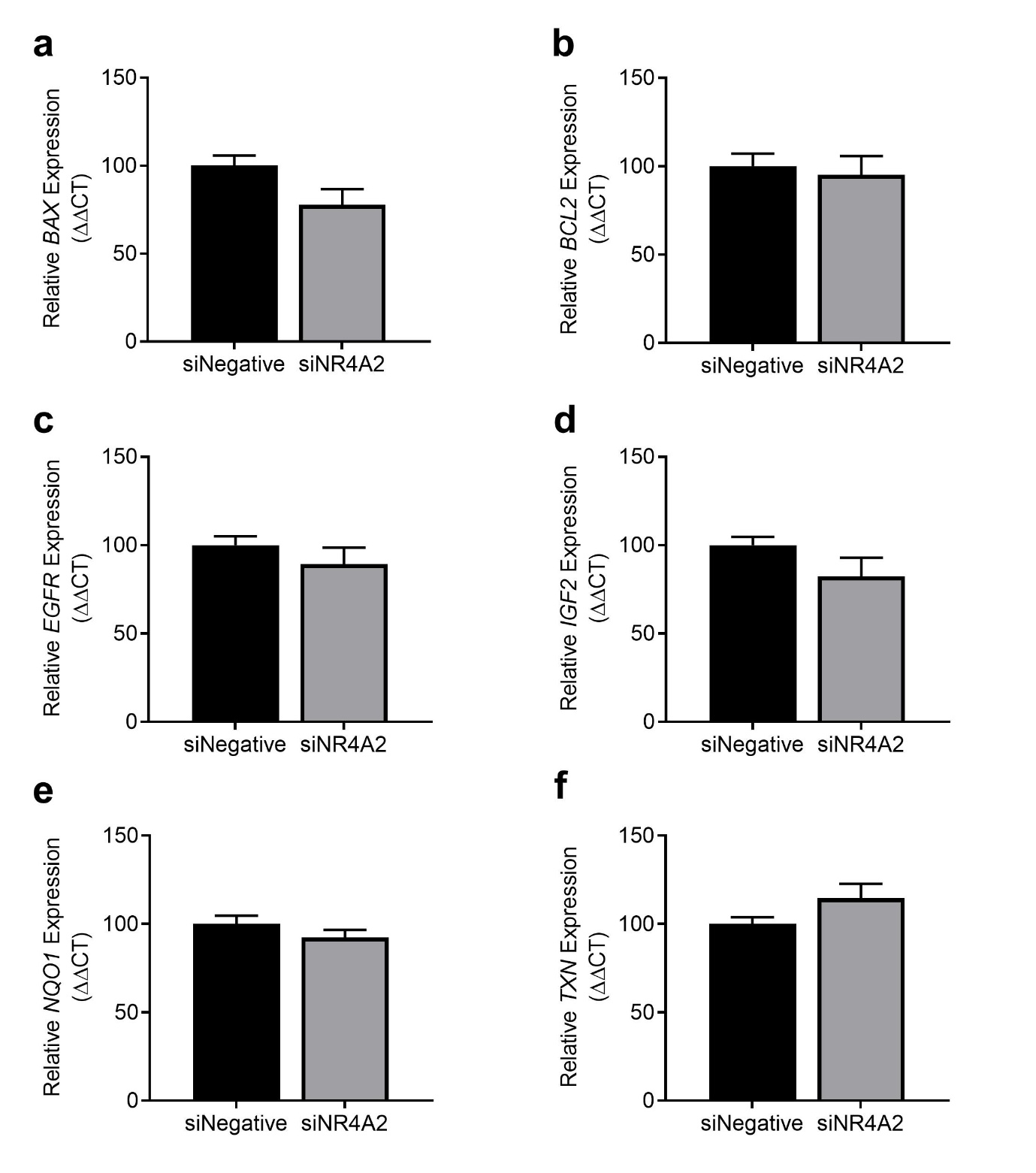


**Supplementary Fig. S5** Effect of *NR4A2* knockdown in primary cytotrophoblasts on expression of genes associated with apoptosis, growth, and oxidative stress under hypoxic (1% O_2_) conditions. Expression was assessed by qPCR. Expression of these genes was not altered with NR4A2 knockdown compared to negative control. Data presented as relative change from control ± SEM. n=3 experimental replicates, with each sample from a different patient. Each experiment was run in triplicate.


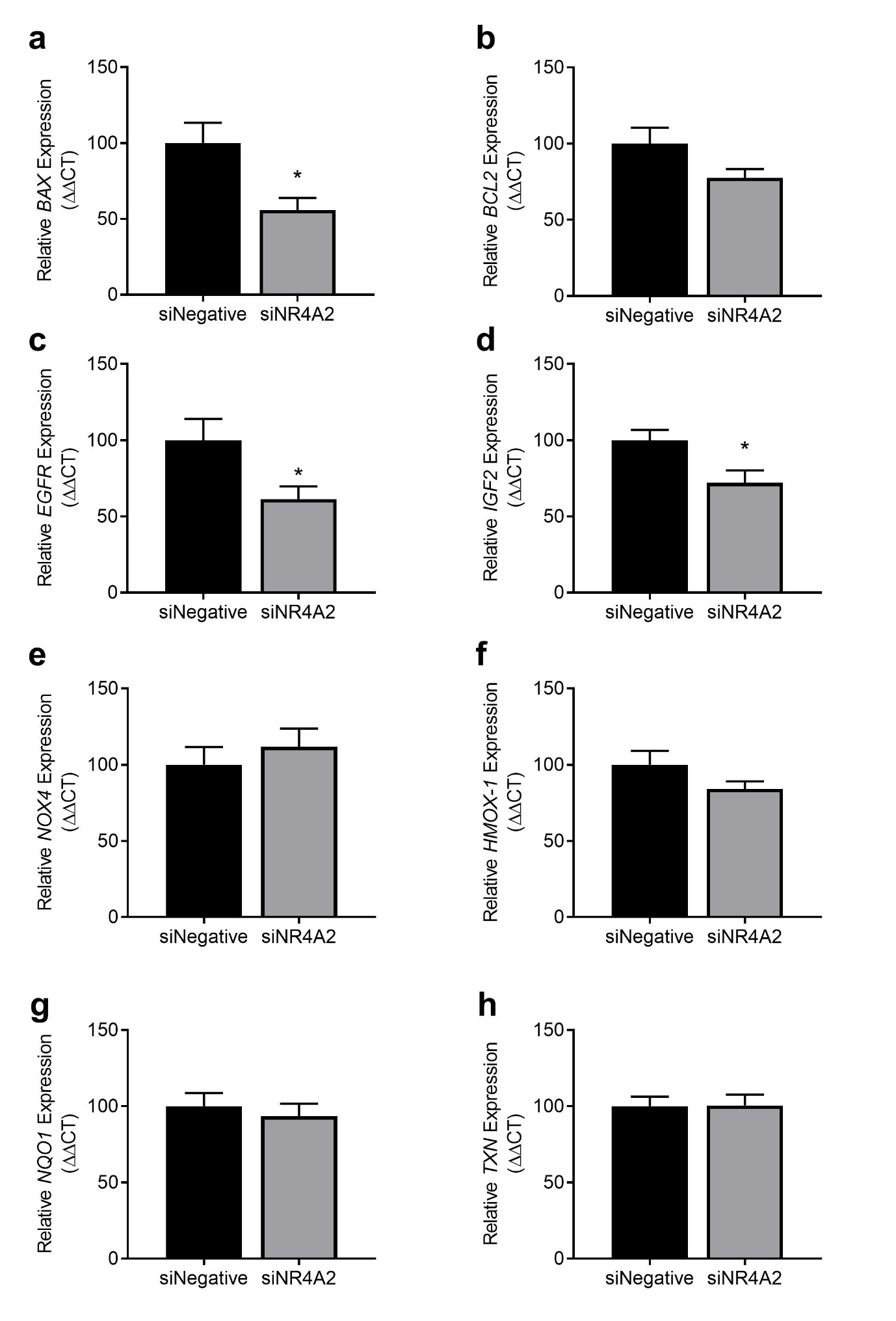

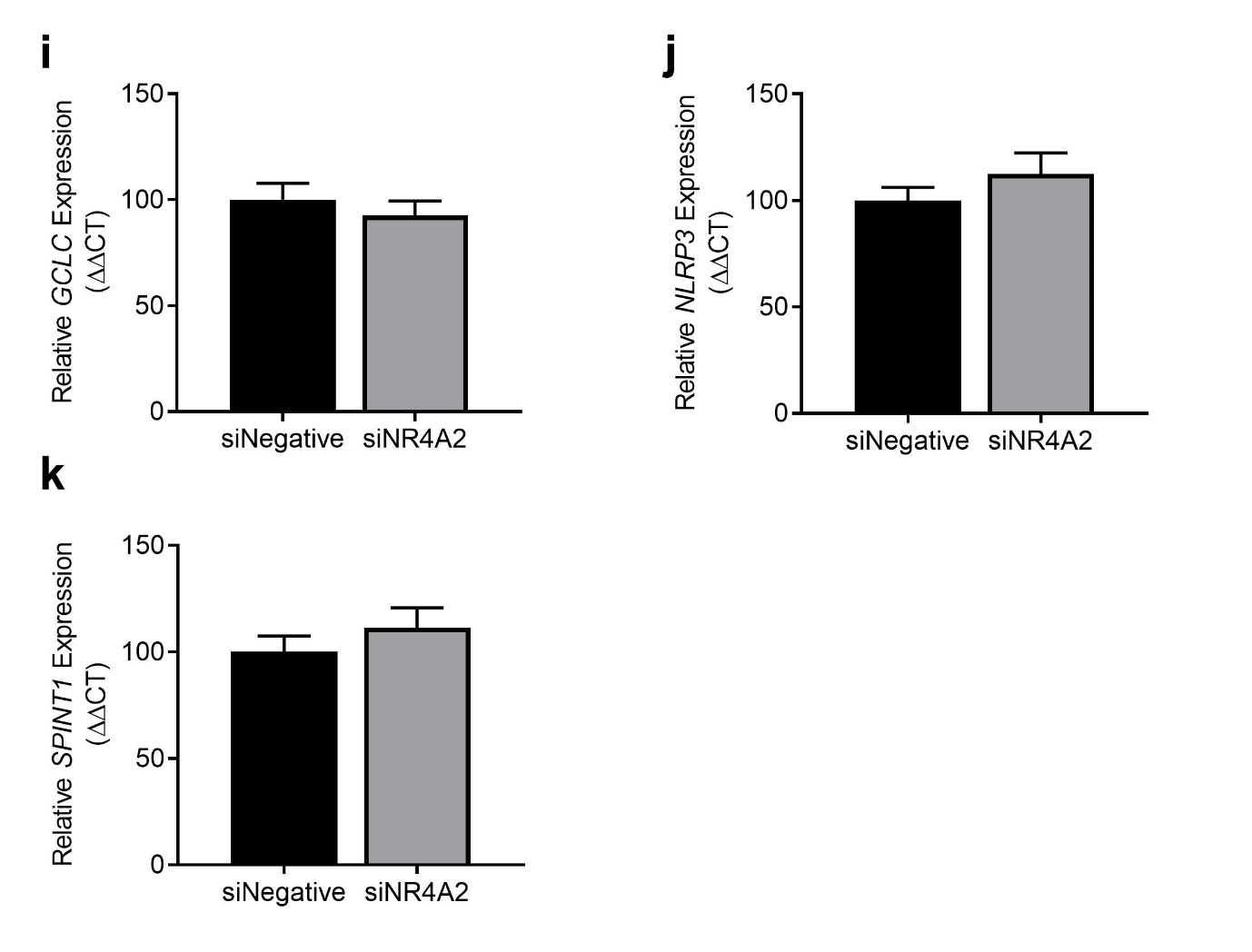


**Supplementary Fig. 6** Gene expression of genes associated with growth, inflammation, apoptosis and oxidative stress under normoxic (8% O_2_) conditions in primary cytotrophoblasts with *NR4A2* knockdown**.** The expression of *BAX*, *EGFR* and *IGF2* is significantly reduced with *NR4A2* silencing compared to negative control. No other genes were significantly altered. Data presented as relative change from control ± SEM. *p<0.05. n=3 experimental replicates, with each sample from a different patient. Each experiment was run in triplicate.
